# Metabolic Heterogeneity Evidenced by MRS among Patient-Derived Glioblastoma Multiforme Stem-Like Cells Accounts for Cell Clustering and Different Responses to Drugs

**DOI:** 10.1155/2018/3292704

**Published:** 2018-02-18

**Authors:** Sveva Grande, Alessandra Palma, Lucia Ricci-Vitiani, Anna Maria Luciani, Mariachiara Buccarelli, Mauro Biffoni, Agnese Molinari, Annarica Calcabrini, Emanuela D'Amore, Laura Guidoni, Roberto Pallini, Vincenza Viti, Antonella Rosi

**Affiliations:** ^1^National Centre for Innovative Technologies in Public Health, Istituto Superiore di Sanità, 00161 Rome, Italy; ^2^Istituto Nazionale di Fisica Nucleare INFN Sez. di Roma, 00185 Rome, Italy; ^3^Department of Oncology and Molecular Medicine, Istituto Superiore di Sanità, 00161 Rome, Italy; ^4^National Center for Drug Research and Evaluation, Istituto Superiore di Sanità, 00161 Rome, Italy; ^5^National Centre for Animal Experimentation and Welfare, Istituto Superiore di Sanità, 00161 Rome, Italy; ^6^Institute of Neurosurgery, Università Cattolica del Sacro Cuore, 00168 Rome, Italy

## Abstract

Clustering of patient-derived glioma stem-like cells (GSCs) through unsupervised analysis of metabolites detected by magnetic resonance spectroscopy (MRS) evidenced three subgroups, namely clusters 1a and 1b, with high intergroup similarity and neural fingerprints, and cluster 2, with a metabolism typical of commercial tumor lines. In addition, subclones generated by the same GSC line showed different metabolic phenotypes. Aerobic glycolysis prevailed in cluster 2 cells as demonstrated by higher lactate production compared to cluster 1 cells. Oligomycin, a mitochondrial ATPase inhibitor, induced high lactate extrusion only in cluster 1 cells, where it produced neutral lipid accumulation detected as mobile lipid signals by MRS and lipid droplets by confocal microscopy. These results indicate a relevant role of mitochondrial fatty acid oxidation for energy production in GSCs. On the other hand, further metabolic differences, likely accounting for different therapy responsiveness observed after etomoxir treatment, suggest that caution must be used in considering patient treatment with mitochondria FAO blockers. Metabolomics and metabolic profiling may contribute to discover new diagnostic or prognostic biomarkers to be used for personalized therapies.

## 1. Introduction

Glioblastoma (GBM) is the most aggressive brain tumor in adults with a median survival of 14 months [1]. Current treatments that include surgery, radiotherapy, and chemotherapy, with temozolomide, are largely unsatisfactory and only achieve a modest prolongation of average patient survival.

As other cancers, GBM displays large heterogeneity among patients with relevant differences in genome, transcriptome, proteome, and metabolome features; in addition, it comprises quite different cell populations in the same patient [2, 3]. Both inter- and intraindividual heterogeneity may cause failure of treatments and relapse. The cancer stem cell hypothesis postulates the existence of a small fraction of self-renewing cells within GBM with stem-like properties (e.g., the capacity of initiating tumor formation *in vivo*) and high resistance to radiation and chemotherapy [4–6]. Indeed, drug resistance of glioma stem-like cells (GSCs) is thought to be responsible for tumor recurrence. Cultivation of GBM cells *in vitro* using specific serum-free conditions facilitates the generation of GSCs [7]. Subclasses of high-grade glioma have been identified based on molecular gene expression [8] that included the proneural (PN), proliferative (Prolif), and mesenchymal (Mes) subtypes. Further studies on expression profiling revealed the presence of two distinct subsets of GSCs: (a) GSf, displaying a full stem-like phenotype, highly tumorigenic and invasive *in vivo*, and expressing an enriched proneural gene expression signature; (b) GSr, with a restricted stem-like phenotype showing expression signatures more similar to commercial cell lines (e.g., T98G) than to original patient tumors [9].

Metabolic reprogramming typical of cellular transformation [10] is gaining importance to identify malignance signatures and offers new approaches to develop tailored therapeutic approaches. Metabolomics and metabolic profiling, as the chemical reflection of a particular phenotype, may contribute to discover new diagnostic or prognostic biomarkers to be used for individualized interventions. Stratifying patients according to molecular biomarker profiles is a key step to manage patient heterogeneity. In previous studies [11, 12], we showed that stratification of GSCs according to their metabolic MRS signals paralleled the genetic profiles typical of GSf/GSr cell lines [9].

Literature data on tumors of different origins [13] make the metabolism of fatty acids (FA) worth of attention in GSCs. Accumulation of FAs mostly as neutral lipids has often been observed in cancer cells *in vitro* and *in vivo* mainly by means of MR techniques, particularly in brain tumors [14]. These lipids, known as mobile lipids (MLs), are mainly originated from triglycerides and may be alternatively (i) arranged in small isotropically tumbling microdomains embedded within the plasma membrane; (ii) stored in cytoplasmic intracellular neutral lipid droplets (LDs), or (iii) extracellularly located in the necrotic core of tumors. These lipids may play a role in cell detoxification and act as a source of energy for new membrane synthesis or as a fuel in fatty acid oxidation (FAO) after lipolysis [15]. Different GSCs show a high heterogeneity of intensities in lipid signals attributed to MLs [16, 17].

In cancer cells, FA synthesis is upregulated due to the accelerated cell proliferation [18, 19]. Studies conducted on glioma cells revealed metabolism with high levels of lipids, suggesting a role for lipid-targeted therapies in these brain tumors [20]. On the other hand, FAO has been suggested as relevant for cell survival [13, 21]. In fact, treatment with etomoxir, a FAO inhibitor through the carnitine palmitoyltransferase 1 (CPT1) pathway, impairs NADPH production and increases reactive oxygen species generation, resulting in ATP depletion, death of human GBM cells, and prolonged survival of grafted mice [22, 23].

The purpose of this study was to characterize the metabolic phenotypes of a large set of GSC lines. Clustering through unsupervised analysis of MR spectral data allowed us to identify three GSC subgroups with different cellular signatures. The existence of subclones in the same line with different metabolic phenotypes was also found, demonstrating intratumoral heterogeneity of GSCs. Cell energy metabolism was analyzed through combined examination of cells and culture media. A prevalence of aerobic glycolysis in cluster 2 lines was observed, while a role for lipids with contribution of mitochondrial FAO is present in some cluster 1 cells. Different responses to treatment with oligomycin and with the FAO inhibitor etomoxir were observed by both MRS and confocal microscopy and were related to GSC heterogeneity. Treatment with etomoxir produced different effects even in GSCs belonging to the same metabolic/genetic signature cluster that may account for differences of response to therapies.

## 2. Methods

### 2.1. Enrollment of Patients, Diagnosis, and Tumor Characterization

Tumor tissue samples were harvested from 44 patients undergoing craniotomy at the Institute of Neurosurgery, Università Cattolica del Sacro Cuore (UCSC), Rome, Italy. All the patients provided written informed consent according to the research proposals approved by the Ethical Committee of UCSC. Patients were eligible for the study if a diagnosis of GBM was established histologically according to the WHO classification.

### 2.2. GSC Isolation, Cell Culture, and Drug Treatment

GSC lines were isolated through mechanical dissociation of tumor tissues and cultured in a serum-free medium supplemented with EGF and basic FGF as described [7]. The neural origin and stemness of cultured cells were assessed by phenotypic and functional characterization [24]. Stem cell marker (CD133 and Sox2) expression was evaluated by flow cytometry with a Canto analyzer (Becton Dickinson, Milan, Italy). To assess clonogenicity, viable cells were seeded at different densities (1-3-10 cells/well) in 96-well plates by cell sorting (FACS Aria, Becton Dickinson). After two weeks, wells with growing clones were enumerated, and results were analysed by Extreme Limiting Dilution Assay (ELDA) software. The *in vivo* tumorigenic potential of GBM neurospheres from 30 out of 37 GSC lines was assayed by intracranial or subcutaneous cell injection in immunocompromised mice. GBM neurospheres were capable to generate a tumor identical to the human tumor both in antigen expression and histological tissue organization. GSC lines were validated by short tandem repeat (STR) DNA fingerprinting. Adult human brain olfactory bulb neural progenitor cells (OB-NPCs) were isolated from surgical specimens as previously described [25].

Cell proliferation was monitored by counting the cells and confirmed by using the Cell Titer-Blue Viability Assay (Promega) and presented as fold change with respect to control at time = 0.

Clones from GSCs were obtained by plating single cells into 96-well plates. After 4 weeks, single clones were mechanically dissociated and replated to expand the culture.

For oligomycin and etomoxir treatments, 2000 cells in exponential growth phase at a density of 2 × 10^4^ cells/ml were dispensed in each well of 96-well plates or in 75 cm^2^ flasks and incubated at 37°C in a 5% CO_2_ atmosphere. After one week of culture, cells were treated in triplicate with 1 *μ*M oligomycin for 24, 48, and 72 hours or with 200 *μ*M etomoxir for 6 hours and then used for MRS experiments.

### 2.3. 1H MRS Cell Sample Preparation

All analyzed cells were removed and washed in PBS and centrifuged at 162 rcf for 3 min. The pellet was suspended in PBS with 20% D_2_O and 2 mM sodium 3-(trimethylsilyl)propionate-2,2,3,3-d_4_ (TMSP) as a frequency standard. A 15 *μ*l aliquot of the suspension was transferred into a 1 mm NMR microtube and centrifuged to obtain a packed cell volume. Conditioned media were collected from cell cultures, added with 20% D_2_O and 2 mM TMSP, and transferred into a 1 mm NMR microtube.

All MRS reagents were purchased from Cambridge Isotope Laboratories, Inc.

### 2.4. Rat Brain Sample Preparation

Male Wistar rats, from Harlan Laboratories, were anesthetized with a mixture of ketamine (90 mg/Kg b.w.) and medetomidine hydrochloride (0.4 mg/kg b.w.) and euthanized by decapitation, and then the brain was removed.

All procedures related to animal experiments and care were performed in accordance with the Legislative Decree n.116/92 [26], which represented the Italian enforcement of the European Directive 86/609/EEC [27] authorized by the Italian Health Ministry (D.M. n. 133/2004-B).

The brain was homogenised using a lancet, suspended in PBS, and centrifuged. The pellet obtained was suspended in PBS with 20% D_2_O and 2 mM TMSP and transferred into the NMR microtube.

### 2.5. Confocal Microscopy and Flow Cytometry Analysis

Nile red-stained samples (50 ng/ml in Hanks' Balanced Salt Solution) were analyzed by confocal microscopy and flow cytometry, in order to visualize the intracellular presence/distribution of lipid droplets and to indirectly quantify their content, respectively [28]. For confocal analysis, cells were deposited on slides and observed with a Leica TSP2 confocal microscopy and the yellow fluorescence was collected with the filter set for fluorescein (450/500 nm band-pass excitation filter, 510 nm centered dichroic mirror, and 528 nm long pass barrier filter). For flow cytometry, cells were analyzed on a LSRII flow cytometer (Becton Dickinson, Franklin Lakes, NJ, USA), with an excitation wavelength of 488 nm, and fluorescence emission was measured at 530 ± 15 and a 575 ± 13 nm corresponding to Nile red green-yellow (FL1) and yellow-gold fluorescence (FL2) arising from MLs, respectively. At least 10,000 cells were counted per analysis. The FL1 and FL2 intensity values were expressed as mean fluorescence channel (MFC).

### 2.6. 1H MRS Measurements

1H MRS experiments were run on a digital Bruker Avance spectrometer at 400.14 MHz, equipped with a 1 mm microprobe. Both one dimensional (1D) and two dimensional correlation spectroscopy (2D COSY) experiments were performed, at T = 298 K.

1D 1H MRS spectra of GSCs and culture media were acquired with a 90° RF pulse, and the number of scans (ns) was equal to 1000 (sufficient to obtain a good signal-to-noise ratio) for cell spectra while ns = 4000 was used for culture media spectra. When indicated, a Lorentzian-Gaussian function was applied in the time domain, before Fourier transformation.

2D COSY spectra were acquired with a 90°-t1–90°-t2 pulse sequence and ns = 32 for cell or ns = 128 for culture media samples. Spectra were acquired as a matrix of 512 × 128 data points in time domain.

### 2.7. Statistical Analysis

Unsupervised agglomerative hierarchical clustering was performed utilizing XLSTAT software, Addinsoft™, version 2012.2.02. Values of log2(FC) ((FC) fold change) resulting from the comparison between the clusters 1a,1b, and 2 were calculated for each metabolite. Student's *t*-test was performed utilizing XLSTAT software, Addinsoft, version 2012.2.02.

## 3. Results

### 3.1. Clustering of GSC Lines

Forty-four GSC lines, derived from the surgical specimens of newly diagnosed GBM patients, and ten subclones, five from GSC line #1 and five from #83, were profiled by MR spectroscopy to identify tumor groups with different metabolisms. Metabolic profiling of the normal rat brain was also performed in order to compare detectability of brain metabolites and their assignments.

Many metabolite signals were detectable in all samples, although with considerable differences of intensity, thus characterizing prevalence of different metabolism.

Spectra (low and high field regions) of normal rat brain and of three GSC lines are shown in Supplementary Figure S1. Relative signal assignments and deconvolutions are presented in Supplementary Table S1 and Supplementary Figure S2.

NAA, GABA, Gln, A (ML cross 2D peak), Myo-I, Asp, Glu, glutathione (GSH), and N-acetylgalactosamine (GalNAc) were quantified in 2D COSY spectra, whereas UDP-hexosamine and tCr in 1D spectra as previously described [16]. Moreover, in the present study, we analyzed intensities of ML and Gly (in 1D spectra) and PC and GPC signals (in 2D COSY spectra). Gly signals could be relevant in view of the key role suggested for Gly in tumors [29, 30]. Neural metabolic signals like NAA or Myo-I were present in cell spectra as well as in those of normal rat brain. The presence of large amounts of ML characterizes a metabolism typical of commercially available cancer cell lines [14].

**Figure 1:**
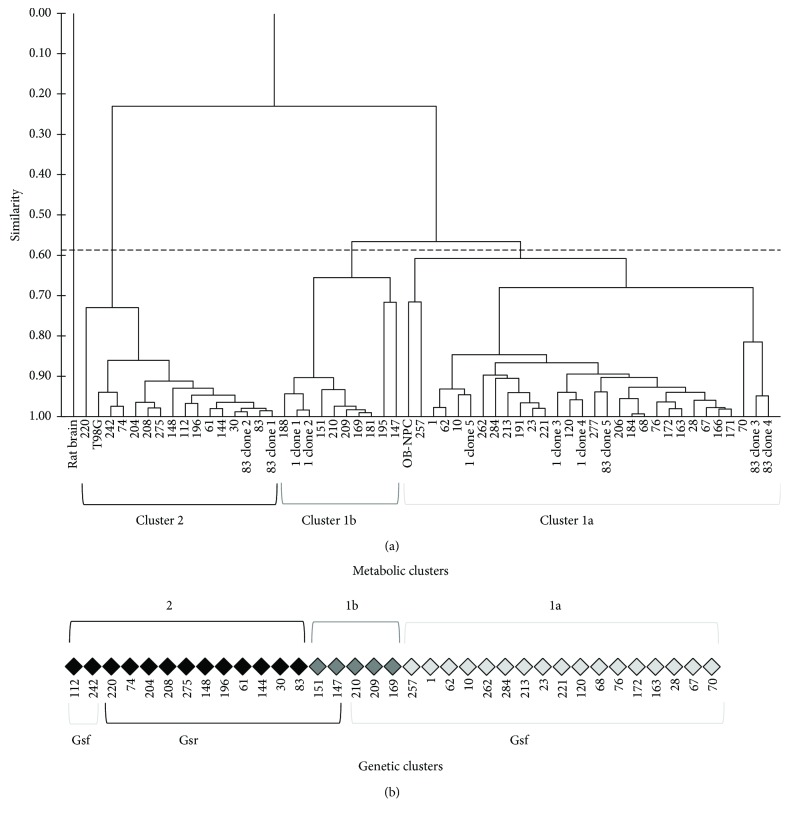
Metabolic clustering of GSCs. (a) Dendrogram resulting from unsupervised cluster analysis of metabolic data from MR spectra of rat brain, forty-four GSCs, OB-NPC, and T98G and five clonal sublines of line #1 and five of line #83. All samples were analyzed in triplicate. Cutting the dendrogram at an appropriate level, analysis allowed to separate tested cell lines into three clusters: 1a, 1b, and 2. Rat brain did not show any similarities with all other lines. (b) Comparison of metabolic and genetic clustering [11, 12].

Unsupervised cluster analysis of MRS signal intensities from spectra of forty-four GSC lines, five clonal sublines of line #1 and five of line #83, T98G cells, and OB-NPC cells, all previously profiled [16], and of normal rat brain was performed. Analysis allowed to separate cell lines into clusters 1a and 1b (with high similarity) and cluster 2. Rat brain did not show any similarities with all other lines (Figure 1(a)).

**Figure 2:**
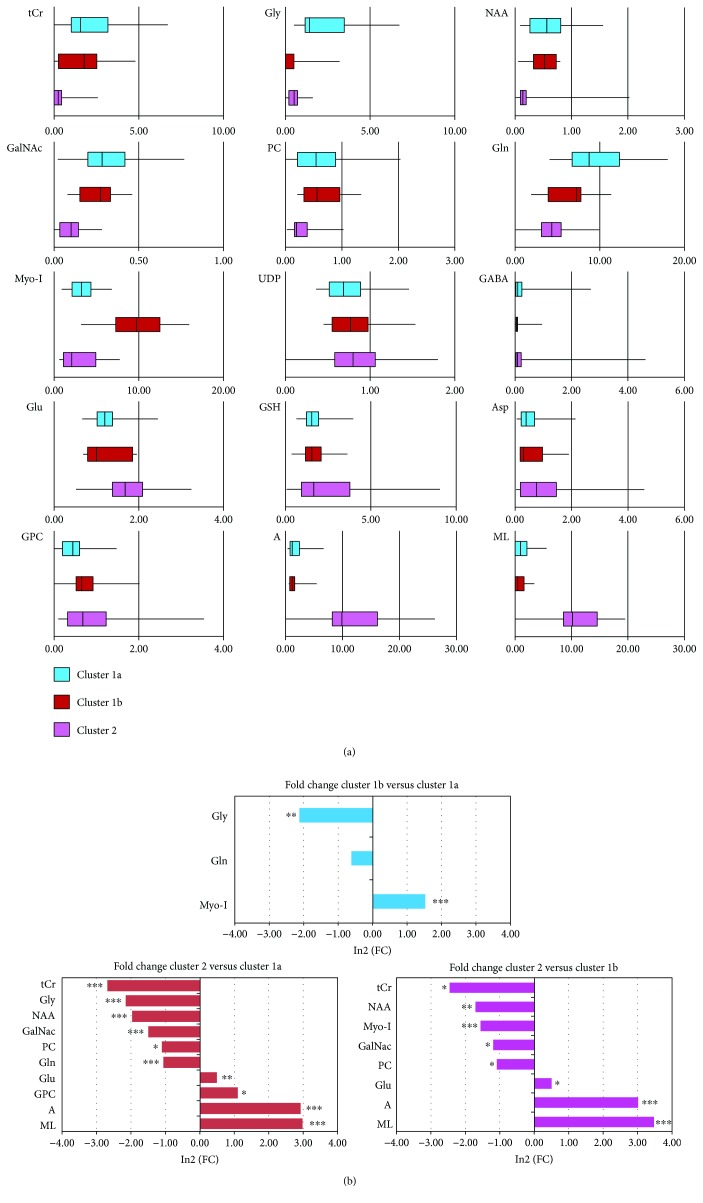
Statistics of metabolic clustering. (a) Box-and-whisker plots of metabolite signal intensities measured in MR spectra from GSC lines belonging to cluster 1a (blue), 1b (brown), and 2 (pink). Intensities of NAA, GABA, Gln, A, Myo-I, Asp, Glu, GSH, PC, and GPC were quantified in 2D COSY spectra and GalNAc, UDP, Gly, ML, and tCr in 1D spectra. (b) log2(FC) ((FC) fold change) of metabolite signal intensities of cluster 1b with respect to cluster 1a, of cluster 2 with respect to cluster 1a, and of cluster 2 with respect to cluster 1b. Only significant changes are reported with ^∗^ *p* < 0.05, ^∗∗^ *p* < 0.005, and ^∗∗∗^ *p* < 0.0005.

Signal intensities of the fifteen metabolites for the three clusters are reported in Figure 2(a) as box-and-whisker plots. Statistically significant differences between metabolite signal intensities from 1a, 1b, and 2 clusters were measured as fold change (FC) (Figure 2(b)).

Clusters 1a and 1b lines were characterized by a prevalent neural fingerprint, with the highest intensities of neural markers NAA and of tCr. Furthermore, cluster 1b when compared to cluster 1a had the highest intensity of the astrocyte marker Myo-I, while cluster 1a showed peaks for Gly and Gln. Cluster 2 lines, with the highest intensity of lipid signals, high GPC, and very low PC, NAA, and tCr signals, were characterized by a prevalent fingerprint of serum-cultured tumor lines (Figure 2(a)).

Interestingly, our previous results on gene expression of 35 GSCs [11, 12] evidenced the presence of two clusters, GSf and GSr, recalling those identified by Schulte et al. [9]. Gene expression data confirmed our MRS metabolic profiling, since cluster 1a was nicely included in GSf cells, whereas clusters 1b and 2 were mostly included amongst GSr cells (Figure 1(b)).

Prevalence of different metabolic profiles assigned subclones of line #1 in cluster 1 and subclones of line #83 both into clusters 1 and 2. This observed intracell heterogeneity could be relevant in patient's response to therapies. The line T98G was in cluster 2 while line OB-NPC was classified in cluster 1a (Figure 1(a)).

### 3.2. Lipids and Lactate in GSC Lines

**Figure 3:**
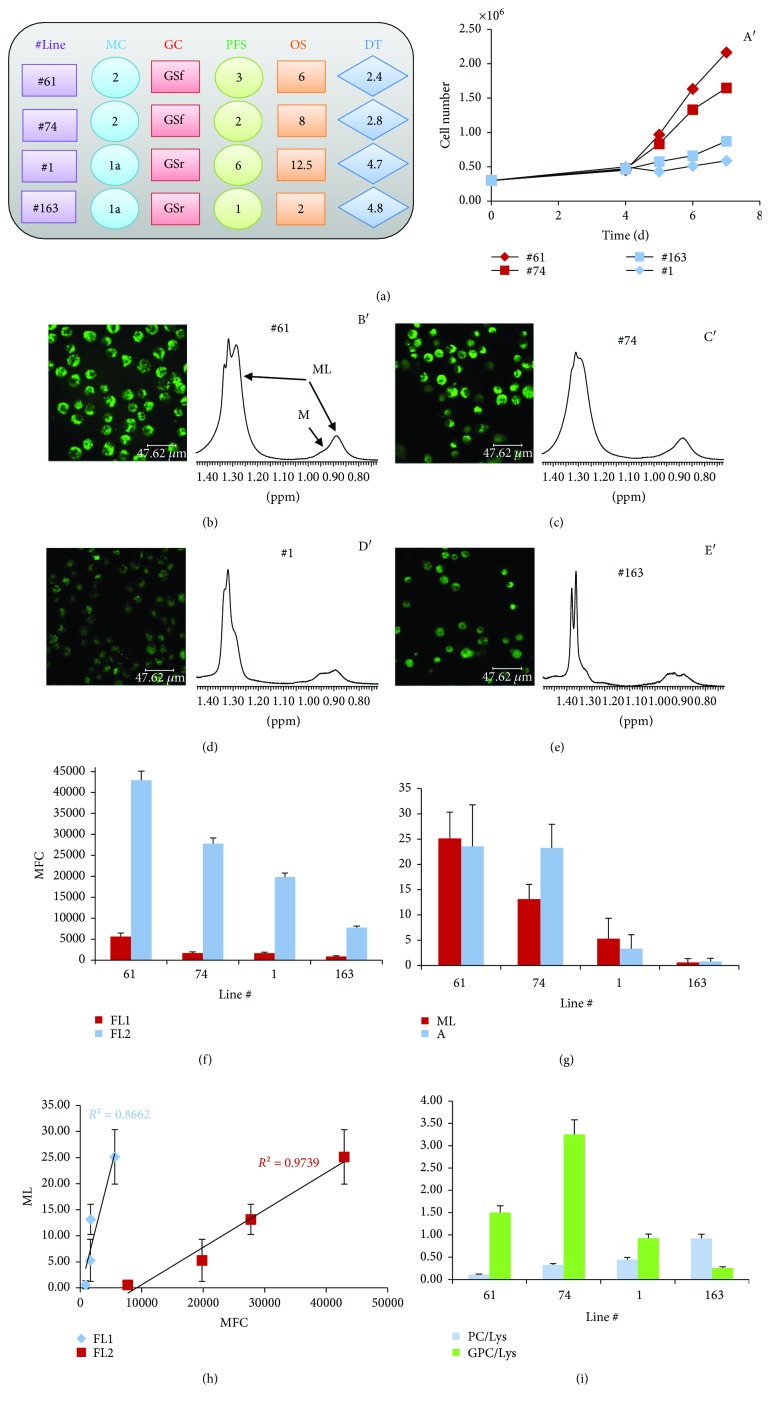
Intracellular lipid droplets are detected by MRS and by ME mainly in GSCs of cluster 2. (a) Characteristics of the selected four GSC lines: metabolic and phenotypic cluster classification; progression-free survival (PFS) and overall survival (OS) of the corresponding patients; doubling time (DT) of GSCs in culture; (A′) in vitro growth curves of the four different GSC lines. Confocal microscopy images (b–e) and mobile lipid (ML) signal region from 1D 1H MR spectra (B′, C′, D′, and E′) of GSC lines #61, #74, #1, and #163, respectively; (f) mean fluorescence values (MFC), FL1 and FL2 channel, from Nile red-stained GSC samples analyzed by flow cytometry; (g) mean values (and SE) of mobile lipid signals from 1D (ML) and 2D COSY (cross peak A) MR spectra (at least three experiments for each GSC line); (h) linear correlation between fluorescence values and ML signal intensities; (i) mean values (and SE) of PC and GPC values as obtained from 2D spectra.

ML signal intensity was one of the parameters that mostly characterized cluster 2 cells compared to cluster 1a and 1b cells. To clarify the origin of ML signals, two GSr-like lines from cluster 2 (lines #61 and #74) and two GSf-like lines from cluster 1a (lines #1 and #163) were examined by MRS, confocal microscopy, and flow cytometry. Lines #1 and #163, although belonging to the same metabolic cluster 1a and to the same GSf-like group [11], were generated from patients with quite different outcomes. Lines #61 and #74 showed higher growth rate than #1 and #163 (Figure 3(a) and A′).

Confocal microscopy and flow cytometry showed that LDs were present in the cytoplasm of all examined lines, but their content was higher in lines #61 and #74 compared to lines #1 and #163 (Figures 3(b)–3(f)). In parallel, higher intensity of ML signals was observed in MRS spectra of lines #61 and #74 (Figures 3 B′, C′, D′, and E′ and 3(g)) as expected for lines belonging to cluster 2. A linear correlation between fluorescence values and ML signal intensities was observed (Figure 3(h)). Moreover, high ML levels paralleled high intensity of GPC catabolite signal (Figure 3(i)).

**Figure 4:**
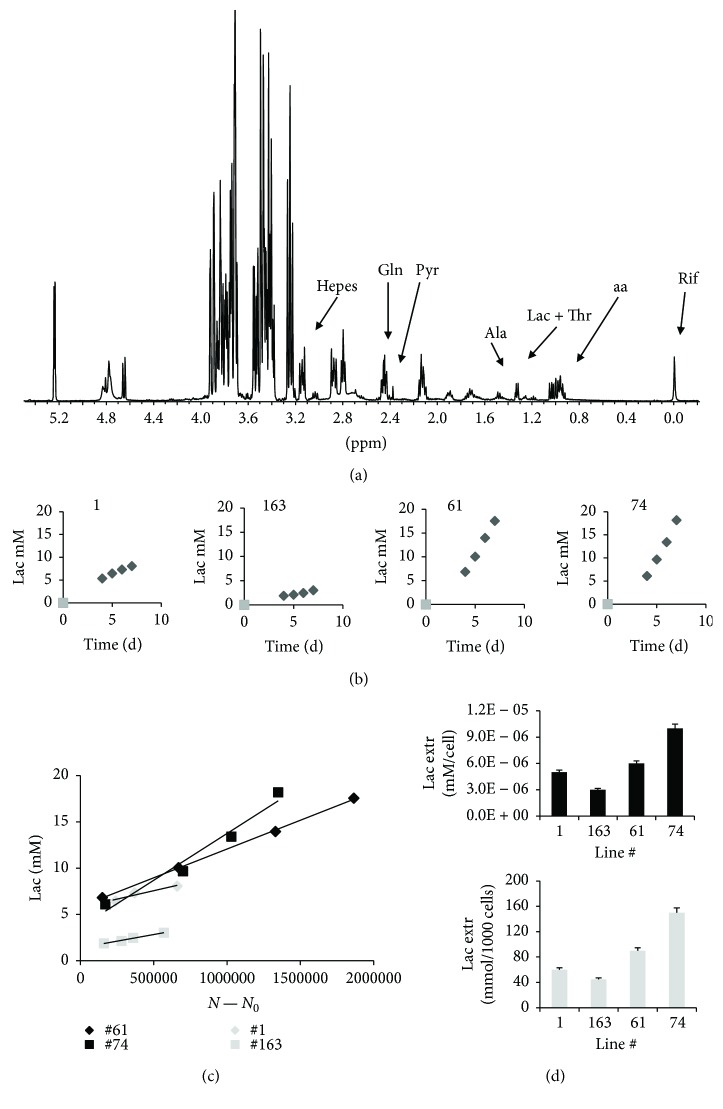
Lactate is differently extruded by different GSCs. (a) MR spectrum of culture medium for line #163, at day 4 after seeding; (b) behavior of Lac signal intensity as a function of time, starting at day 4 after seeding; (c) excreted lactate from the selected GSC lines as a function of the number of cells (*N* − *N*_0_, where *N*_0_ is the number of seeded cells); the goodness of the linear fit, *R* ^2^, is in the range 0.96–0.99 for all the lines. (d) reports the rate of lactate extrusion (mM/cells) and mmoles of lactate for 1000 cells, respectively, for all four lines. Deconvolution of Lac signal regions is reported in Figure S2C.

Lactate (Lac) extrusion was examined in culture media spectra (Figure 4(a)) to evaluate the correlation of clustering with energy metabolisms. Lac production increased with time in culture for all the analyzed lines (Figure 4(b)). Linear Lac extrusion, as a function of cell number during cell growth, was observed in the four lines, being higher in lines #61 and #74 compared to lines #1 and #163 (Figures 4(c) and 4(d)).

A connection between glycolytic metabolism and lipid droplet content is suggested by comparison of data from lines #1 and #163 with lines #61 and #74. Moreover, Gln consumption increased as a function of cell number during cell growth (data not shown) for all examined lines, thus confirming that these cells were all Gln addicted.

### 3.3. Energy Requirements of GSC Lines

Different energy sources may be exploited by cells from clusters 1a and 2, possibly including mitochondrial FAO. Thus, the role of FAO in #61, #74, #1, and #163 cell lines was investigated.

The effects of oligomycin, a mitochondrial ATPase inhibitor, were firstly examined. Oligomycin had no effect on cell proliferation in lines #61 and #74 up to 24 and 48 hours of treatment while a proliferative arrest was detected in lines #1 and #163. For longer time intervals, oligomycin was toxic to all cell lines (Figure S3A). MR spectra showed the presence of saccharopine, an intermediate in the Lys degradation pathway activated by the oligomycin treatment in line #163 (Supplementary Figure S3B), in agreement with previous data [17], and in lines #61 and #74. On the contrary, saccharopine was almost undetectable in line #1-treated cells (data not shown).

**Figure 5:**
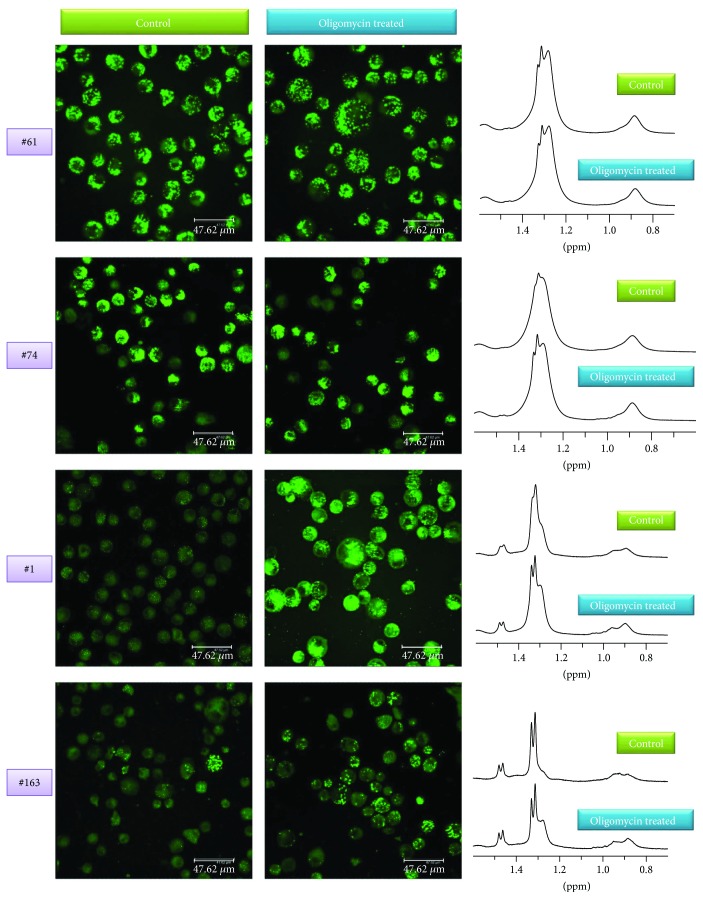
Analysis of the effects induced by oligomycin treatment. Confocal microscopy images and ML signal region from 1D 1H MR spectra of control and oligomycin-treated GSC lines #61, #74, #1, and #163.

**Figure 6:**
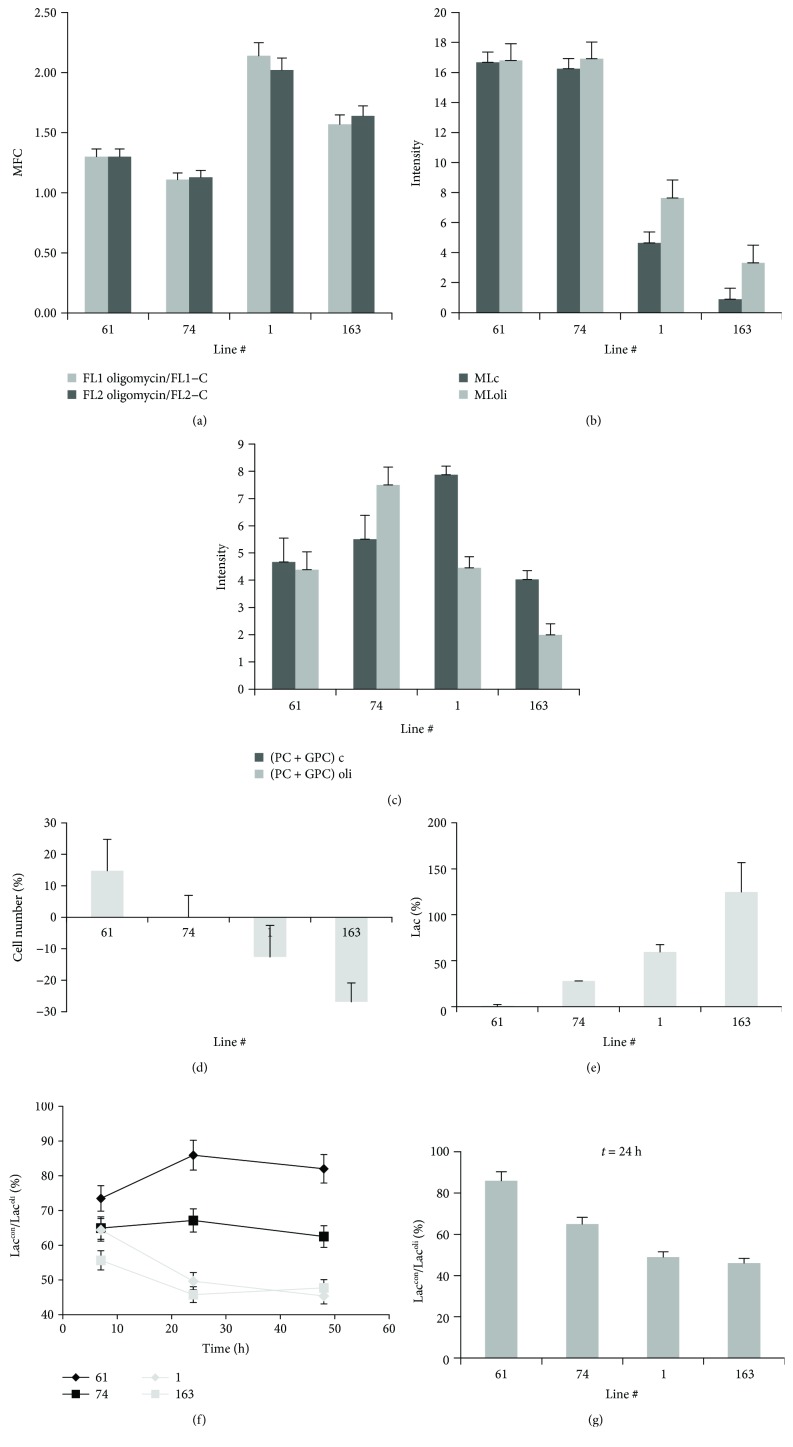
Analysis of the effects induced by oligomycin treatment. (a) Ratios of fluorescence signal values of treated samples (FL1 and FL2 oligomycin) and those of control ones (FL1 and FL2-C). Trend of ML (b) and PC + GPC (c) signal intensities from 1D spectra of control and oligomycin-treated #61, #74, #1, and #163 line samples. Percentage variation of cell number (d) and lactate signal intensity, measured with respect to cell number [(Lac/N)_oli_ − (Lac/N)_con_]/(Lac/N)_con_, after 24 h oligomycin treatment (e) Representative spectra of line #163 after treatment are reported in Figure S3C. (f) Ratio of lactate signal intensity of control (Lac^con^) and olygomicin-treated samples (Lac^oli^) as a function of time. (g) Values after 24 hours of treatment are reported for all the analyzed lines.

Confocal microscopy revealed an increase of LDs after oligomycin treatment in all cell lines even if at different extent (Figures 5 and 6(a)) that matched ML signal intensity variations in MR spectra (Figures 5 and 6(b)).

Oligomycin induced an increase of lipid signal intensities in cluster 1a lines and had no effect on those in cluster 2. At the same time, the intensity from coalescent 1D signal spectra of PC + GPC decreased in lines #1 and #163 and increased or remained unaltered in the other two lines (Figure 6(c)). A sharp decrease of glucose (Glc) signal was observed in lines #1 and #163 being in the latter associated with a Myo-I increase (not shown).

Oligomycin treatment also induced a variation both of cell number (Figure 6(d)) and of lactate extrusion in culture medium (Figure 6(e)), cell number decreased in lines #1 and #163, lightly increased in line #61, and remained unchanged in line #74 (Figure 6(d)), while lactate signal intensity increased more in lines #1 and #163 compared to lines #74 and #61 (Figure 6(e)). The values of the Lac^con^/Lac^oli^ ratio were stable after 24 hours of treatment (Figures 6(f) and 6(g)) for all the analyzed lines and can be used as an indication of the Warburg effect entity [31].

**Figure 7:**
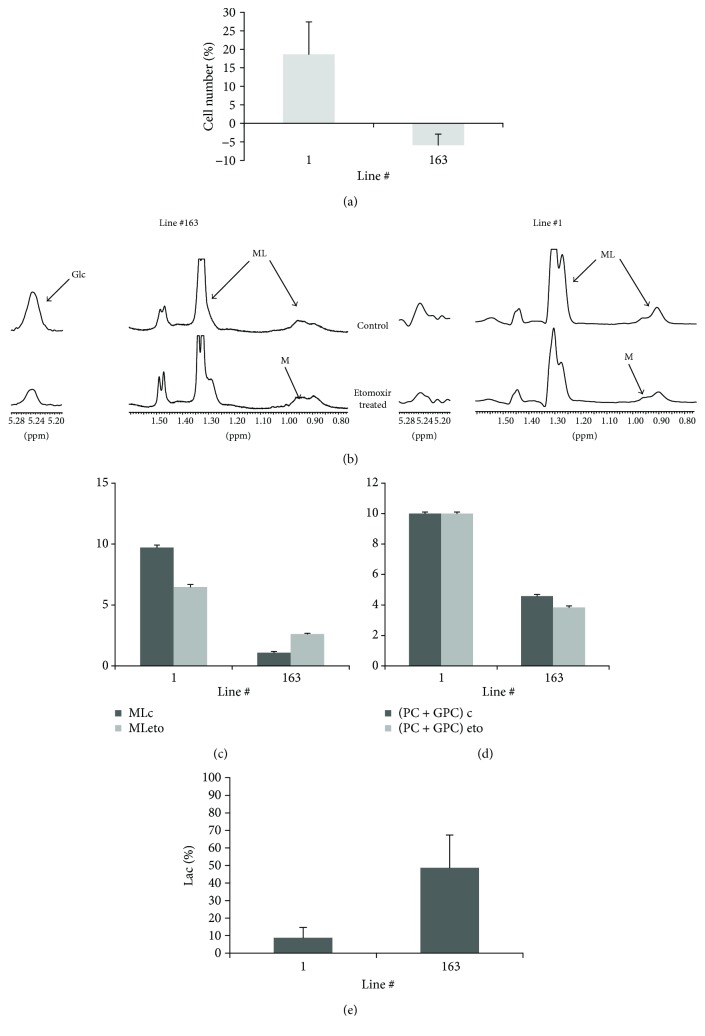
Analysis of the effects induced by etomoxir treatment. (a) Changes in cell number percentage induced by 7-hour etomoxir treatment in cell lines #1 and #163. (b) Effects of etomoxir treatment on ML and Glc signals in 1D MR spectra of lines #1 and #163. Trend of ML (c) and PC + GPC (d) signal intensities from 1D spectra of control and etomoxir-treated #1 and #163 line samples. (e) Percentage variation of lactate signal intensity measured compared to cell number ([(Lac/N)eto (Lac/N)con]/(Lac/N)con) 7 h after etomoxir treatment.

A substantial contribution of FAO to energy requirements is present in lines #1 and #163, different from lines #61 and #74 characterized by high ML signals and LDs. For this reason, we examined the effect of etomoxir on the metabolism of lines #1 and #163 that showed increased lipid signals after oligomycin treatment. Mild etomoxir treatment induced an increase of cell number in line #1 and a nonsignificant proliferative slowdown of #163 cells (Figure 7(a)). Concurrently, a ML signal intensity decrease in line #1 and an increase in line #163 cell spectra were observed after treatment (Figures 7(b) and 7(c)), while Glc signal intensity decreased in spectra of both cell lines (spectra in Figure 7(b)). In parallel, intensities of PC + GPC signals decreased only in line #163 remaining unaltered in line #1 spectra (Figure 7(d)). The decrease of ML in etomoxir-treated line #1 cells was paralleled by a decrease of LDs as evidenced by both confocal and flow cytometric analyses (data not shown). Lactate extrusion in culture media mostly increased in line #163 after treatment (Figure 7(e)).

Finally, oligomycin induced a fumarate signal increase, in both line #1 and #163 spectra, while etomoxir treatment increased fumarate only in line #163 spectra (Supplementary Figure S4).

## 4. Discussion and Conclusions

The hypothesis of the existence of cancer stem cells within tumors has gained prominence because of an increasing amount of evidence, starting from leukemia studies. According to this theory, cancer growth is dependent on a subpopulation of cancer cells that behaves similarly to stem cells present in normal tissues. Metabolomics, as study of metabolites that mirror cell phenotypes, may help in clarifying the critical role of these cells in the generation and progression of cancer as well as in developing therapeutic strategies. Cell metabolism can be pursued by MRS analysis of cell metabolites, including those excreted in culture-conditioned medium.

### 4.1. Metabolic Clustering of GSCs

Stratifying patients on molecular biomarker profiles is a key step to manage interpatient heterogeneity. In this study, we characterized the phenotypes of GSCs from a cohort of 44 GBM patients looking at metabolic markers obtained by MRS. All cell lines displayed a metabolism of neural systems, as highlighted by neural markers NAA and Myo-I. Furthermore, a generic tumor phenotype, characterized by large amounts of ML [14], was also found in these cells. The prevalence of one out of the two metabolisms allowed us to identify two main clusters: clusters 1, including two very closely related subclusters, with a prevalent neural metabolism and cluster 2 with a prevalent metabolism typical of commercial tumor cell lines. In line with this finding, OB-NPC normal progenitor line is clusterd in cluster 1a, while the commercial tumor line T98G belongs to cluster 2.

According to previous results [11, 12], a correlation between the proneural-enriched lines displaying a full stem-like phenotype (GSf lines) and the lines of cluster 1a + 1b was also found. The nonprevalent neural metabolism in GSCs of cluster 2 makes this group more similar to lines with a GSr phenotype. This suggests both that the resemblance between GSC lines and tumors is associated with different degrees of aggressiveness [11] and that in these cells the metabolic rewiring is mainly genetically driven.

The present analysis has shown that lines originating from the same tumor may include subclones belonging to different clusters. This was the case of line #83, classified in cluster 2, which included three subclones out of five belonging to cluster 1. This is a first indication that intratumoral heterogeneity is also present at the metabolic level.

Cell culture media analysis allowed to further characterize lines of different clusters on the basis on their energy metabolic requirements. All examined GSC lines use aerobic glycolysis even if lines #1 and #163 of cluster 1, at lower extent, indicated lower lactate extrusion. Treatment with oligomycin confirmed that cells belonging to cluster 2 mainly rely on aerobic glycolysis, similarly to commercial tumor cells, while GSCs of cluster 1 have a prevalence of OXPHOS, but switch to aerobic glycolysis when challenged, in agreement with the literature data [32].

Phosphoproteomic results are in agreement with the prevalence of aerobic glycolysis in lines #61 and #74 of cluster 2. In fact, the GSr cluster, containing lines #61 and #74, showed upregulation of AKTpS473 phosphorylation that stimulates aerobic glycolysis, compared to cell lines of GSf cluster [33].

### 4.2. Fatty Acid Oxidation

The ML signals are bound to the presence and amount of LDs as indicated by the correlation between flow cytometry and MRS data before and after olygomicin and etomoxir treatments. Our results highlight the physiological relevance of LDs as lipid metabolic reprogramming in cancer cells, matter which is now under extensive examination [33]. De novo FA synthesis (FAS) is the preferred mechanism in cancer cells [19]. Particularly, in glioma cells, approximately 60% of carbon skeletons from glucose are used for de novo FAS, and this was taken as an indication that aerobic glycolysis supports metabolism of fatty acids [34]. The presence of high lipid content in lines #61 and #74 and at minor extent in lines #1 and #163 is in agreement with this hypothesis.

Moreover, our results on the effects of metabolic inhibitors open new perspectives on the role of FA oxidation (FAO) in these cells. The preferred oxidation process in cells is *β*-oxidation that occurs in mitochondria, where it acts as a fuel for cell growth, and in peroxisomes for very long FA chain shortening. FAO is an important candidate for energy supply in cancer cells, now even emerging as a therapeutic target in cancer [13].

In our view, the high lipid amount stored as droplets plays a role in FAO of some cells. LDs may be associated with mitochondria in many cells by the Perilipin protein family [35, 36]. It has been shown in cells such as cardiomyocytes that a direct flow of fatty acids through these proteins from neutral lipid stores to the mitochondrial matrix takes place for *β*-oxidation to meet the cell energy requests [37]. This mechanism is confirmed by the observation that inhibition of *β*-oxidation leads to lipid droplet accumulation after MYC inhibition in NB cells [38]. Moreover, under cell starvation, FAs provide cellular energy by mobilizing and moving LDs into mitochondria in mouse embryonic fibroblasts [39] and LDs supply lipids for mitochondrial *β*-oxidation in VERO fibroblasts [40].

Present data showed higher accumulation of intracellular LDs when mitochondrial activity was impaired in response to oligomycin treatment in lines #163 and #1, both from cluster 1a, and characterized by lower aerobic glycolysis. In these two lines, the oligomycin treatment induced proliferation arrest, indicating the important role played by TCA cycle.

In line #163, cytoplasmic lipids increased after treatment with etomoxir that blocks CPT1, transporter of long FAs into the mitochondrial membrane, thus affecting FAO. Increased LDs observed after mild etomoxir treatment in line #163 reinforce the hypothesis that FAO supports energy requirements of this cell line. This result expands previous evidence on FAO inhibition obtained under metabolic stress [41] or by MYC inhibition [38]. The concomitant lactate increase and glucose decrease can be attributed to the switch to aerobic glycolysis when energy from FAO is lacking. This increase of glycolytic activity is likely to be an adaptive mechanism to counteract any decrease in ATP production similar to what was observed after oligomycin treatment, when mitochondria metabolism was impaired.

### 4.3. Drug Treatment

In line #1, etomoxir treatment induced ML and LD decrease and proliferation increase differently from line #163 suggesting that the storage lipids are more utilized for membrane synthesis. The different responses to etomoxir treatment showed by the two low lipid-rich cell lines are difficult to explain. Both lines, in fact, belonging to the same metabolic and genetic groups, were characterized by low Warburg and by ML and LD increase after mitochondrial disfunction by oligomycin.

After etomoxir treatment of line #1, TCA cycle is not massively altered, as indicated by no increase of fumarate signal intensity, contrary to what happens in this line after oligomycin treatment and in line #163 after both treatments. On the other hand, glucose decrease together with almost absent changes of lactate extrusion in line #1 would be suggestive of an increase of mitochondrial glycolysis to meet energy requirements necessary for proliferation increase. FAs may then be used for membrane synthesis sustaining accelerated proliferation.

A major metabolic difference between the two lines is that line #1, different to line #163, showed almost absent saccharopine formation after oligomycin treatment and belongs to one of the two GSC groups characterized by low/absent adipate content [17]. These two groups were found to be characterized by different patients' survival in the sense that patients with higher adipate showed shorter survival [17]. Recently, high adipate content in serum of patients with ovarian cancer was suggested as a marker of poor patient survival, although a definitive explanation was not given [42]. Involvement of *ω*-oxidation, blockage of peroxisomes, and defective activity of succinic dehydrogenase (SDH) were all indicated as possible sources of the anomalous adipate increase. In line #163, adipate mostly derived from lysine catabolism through mitochondria as blockage of this catabolic pathway by oligomycin leads to formation of the intermediate saccharopine while this molecule is almost lacking in line #1 after the same treatment. This could indicate a peroxisome involvement in the lysine degradation, but peroxisomal contribution to FAO is not likely due to failure of lipid increase in line #1 after etomoxir treatment. On the other hand, succinic aldehyde, final product of SDH, is present in MR spectra of both lines #1 and #163 (not shown).

A further hypothesis to explain differences between lines #1 and #163 could be derived by a different presence of the CPT1 isoforms (a, b, and c) [43, 44]. Recent data evidenced expression of all the three isoforms in glioma with their deregulation in GBM [45]. Tumor cells of different origins expressing CPT1 showed increased FAO [46–48], but cancer cells lacking CPT1c produced less ATP and were more sensitive to metabolic stress [41]. Prevalence of CPT1c with its nonsignificant role in fatty acid oxidation may explain the behavior of line #1.

A different role for CPT1 in line #1 and line #163 could be, therefore, envisaged by present data as well as a role for the adipate source. Although further studies are necessary to clarify this point, the relevant result is that not all GBM cells behave in the same way after treatment that impairs FAO. Caution must therefore be used in considering patient treatment with etomoxir or other mitochondria FAO blockers. Inhibition of CPT1 by etomoxir is proven to suppress cancer growth in glioma cells [22], and etomoxir treatment induced a decrease of proliferating cells in primary-cultured cells isolated from human glioma grown under serum-free conditions and prolonged survival in a syngeneic mouse model of malignant glioma [23]. Present results, by implying the use of fatty acids as an energy source for some aggressive glioma, would confirm FAO inhibition as a possible therapeutic strategy in selected patients.

## Abbreviations

CPT1: Carnitine palmitoyltransferase 1
FAO: Fatty acid oxidation
FC: Fold change
GalNAc: N-acetylgalactosamine
GBM: Glioblastoma multiforme
GSCs: Glioma stem-like cells
GSf: Glioblastoma-full stem-like phenotype
GSr: Glioblastoma-restricted stem-like phenotype
LD: Lipid droplets
ML: Mobile lipids
MRS: Magnetic resonance spectroscopy
OB-NPC: Neural progenitor cells from olfactory bulb
OS: Overall survival
OXPHOS: Oxidative phosphorylation
PFS: Progression-free survival
SDH: Succinic dehydrogenase.

## Supplementary Materials

Supplementary Material Figure S1, Table S1, and Figure S2: spectra (low and high field regions) of normal rat brain and of three GSC lines together with relative signal assignments and deconvolutions. Figure S3A: fold change of cell number, examined by Cell Titer Blue Viability Assay, for #61, #74, #1, and #163 lines after oligomycin treatment. Figure S3B and Figure S3C: spectra of saccharopine and lactate signal regions from control and oligomycin-treated #163 line. Figure S4: fumarate signal from line #163 and line #1 spectra after oligomycin and etomoxir treatments. More details about acquisition and processing of NMR data are reported in Supplemental Experimental Procedures.
