# Supplementary material for: Metabolic Heterogeneity Evidenced by MRS among Patient-Derived Glioblastoma Multiforme Stem-Like Cells Accounts for Cell Clustering and Different Responses to Drugs
Source: Stem Cells Int. 2018 Feb 18;2018:3292704. doi: 10.1155/2018/3292704 (PMC5835274; doi:10.1155/2018/3292704)
Supplement: Supplementary Material — Figure S1, Table S1, and Figure S2: spectra (low and high field regions) of normal rat brain and of three GSC lines together with relative signal assignments and deconvolutions. Figure S3A: fold change of cell number, examined by Cell Titer Blue Viability Assay, for #61, #74, #1, and #163 lines after oligomycin treatment. Figure S3B and Figure S3C: spectra of saccharopine and lactate signal regions from control and oligomycin-treated #163 line. Figure S4: fumarate signal from line #163 and line #1 spectra after oligomycin and etomoxir treatments. More details about acquisition and processing of NMR data are reported in Supplemental Experimental Procedures. [file 3292704.f1.docx]

**Figure S1**

**Myo-I**

*(ppm)*

*3.53*

*3.57*

*3.61*

*3.65*

**a**

**Gly**

*(ppm)*

*0.8*

*1.0*

*1.2*

*1.4*

*1.6*

*1.8*

*2.0*

*2.2*

*2.4*

*2.6*

*2.8*

*3.0*

*3.2*

*3.4*

*3.6*

*3.8*

*4.0*

*0.6*

*(ppm)*

*6.8*

*7.2*

*7.6*

*8.0*

*8.4*

*8.8*

**NAA**

**Asp**

**Glu**

**GABA**

**NAA**

*(ppm)*

*0.8*

*1.0*

*1.2*

*1.4*

*1.6*

*1.8*

*2.0*

*2.2*

*2.4*

*2.6*

*2.8*

*3.0*

*3.2*

*3.4*

*3.6*

*3.8*

*4.0*

*0.6*

**Gln**

**GSH**

**Glu**

**b**

**ML**

**UDP**

*(ppm)*

*6.8*

*7.2*

*7.6*

*8.0*

*8.4*

*8.8*

*(ppm)*

*0.8*

*1.0*

*1.2*

*1.4*

*1.6*

*1.8*

*2.0*

*2.2*

*2.4*

*2.6*

*2.8*

*3.0*

*3.2*

*3.4*

*3.6*

*3.8*

*4.0*

*0.6*

*(ppm)*

*6.8*

*7.2*

*7.6*

*8.0*

*8.4*

*8.8*

**GalNAc**

**Myo-I**

**c**

*(ppm)*

*6.8*

*7.2*

*7.6*

*8.0*

*8.4*

*8.8*

*(ppm)*

*0.8*

*1.0*

*1.2*

*1.4*

*1.6*

*1.8*

*2.0*

*2.2*

*2.4*

*2.6*

*2.8*

*3.0*

*3.2*

*3.4*

*3.6*

*3.8*

*4.0*

*0.6*

**d**

**Gly**

**Gln**

**GalNAc**

**tCr**

**Figure S1.** 1D 1H MR spectra (low and high field region) from representative samples of: normal rat brain (A); GSC lines #61 (B), #169 (C) and #23 (D). Metabolite signals are labelled; signal assignment was performed according to Guidoni (Guidoni et al., 2014). A Lorentzian–Gaussian function was applied for resolution enhancement, LB =10 and GB = 3 for low field and LB =10 and GB = 15 for high field regions, respectively. Neurotransmitters glutammate (Glu), gamma-Aminobutyric acid (GABA), aspartate (Asp) and glycine (Gly), besides the brain marker N-acethyl aspartate (NAA) were clearly visible in 1D spectra of rat brain sample (A). ML signals were very intense in the spectrum of line #61 (B). Spectrum from line #169 (C) showed intense Myo-inositol (Myo-I) signals while spectrum from line #23 was characterized by high Gly, total Creatine (tCr) and glutamine (Gln) signal intensities (D).

**Figure S2**

**Cells**


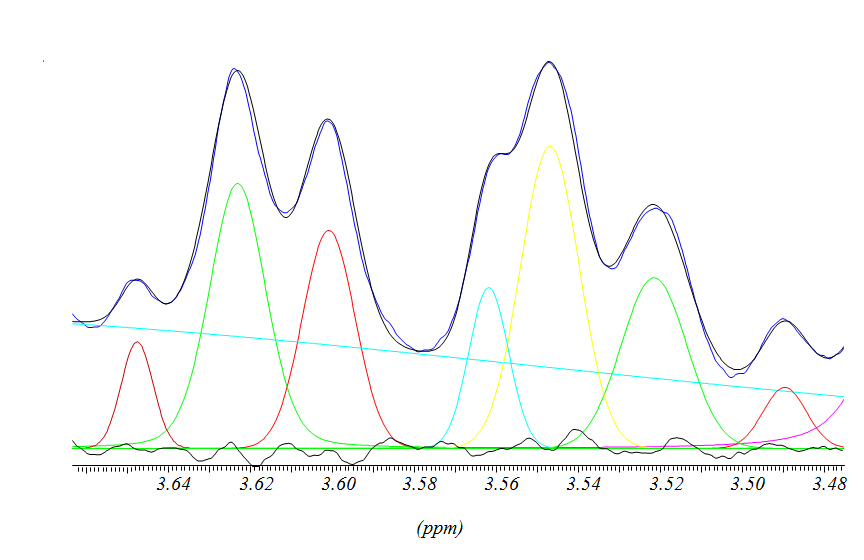


**Gly**

**Myo-I**

**Myo-I**

**B**


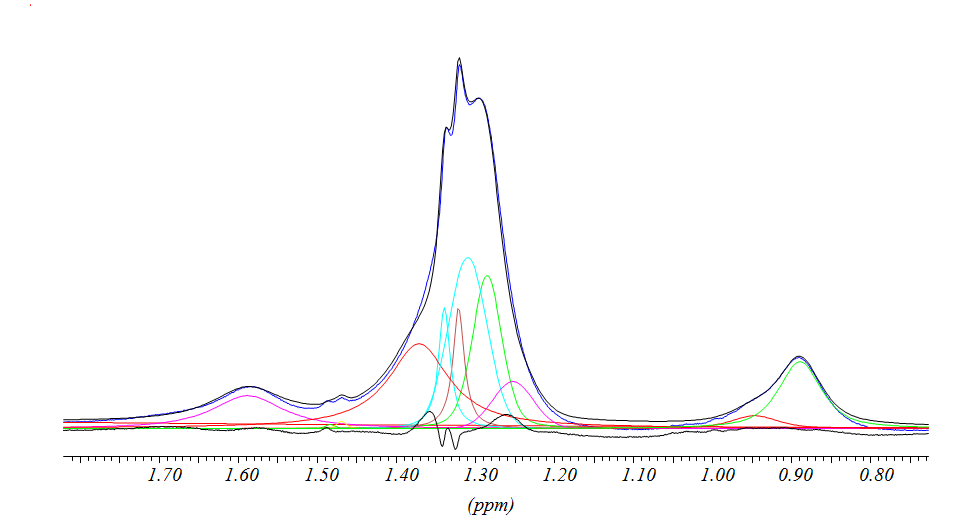


**ML1+MM1**

**MM2**

**MM3**

**ML2**

**ML3**

**Lac1, Lac2**

**ML4**

**A**

**C**

(ppm)

3.76

3.68

3.60

3.52

3.44

(ppm)

4.40

4.32

4.24

4.16

4.08

4.00

**PC**

**GPC**

**Culture media**

*(ppm)*

*1.308*

*1.314*

*1.320*

*1.326*

*1.332*

*1.338*

*1.344*

*1.350*

*1.356*

**Lac2**

**Lac1**

**Thr2**

**Thr1**

**D**

**Figure S2.** Deconvolution of : (A) ML and Macromolecule (MM) and (B) Gly and Myo-I signal regions from a representative spectrum of line #61. ML signal intensity in 1D spectra is proportional to methylene groups of saturated FA chains while the cross peak A from 2D spectra is proportional to the number of saturated and omega-6 unsaturated FA chains as it arises from coupling of terminal methyl and methylene groups. (Jain et al., 2012). (C) 2D COSY GPC and PC spectral region of the #61 line spectrum. PC and GPC signals are strongly related to cell arrest due to confluence or nutrient deprivation, so care was taken by comparing cells in the growth logarithmic phase. (D) Deconvolution of Lactate and Threonine signal region from a representative spectrum of culture medium of line #61.

**Table S1**

| Cells |  |  |
| --- | --- | --- |
| 1D | **δ (ppm)** | **Δν (Hz)** |
|  | **High field** |  |
| ML1+MM1 | 0.89 | 26 |
| MM2 | 0.94 | 28 |
| MM3 | 1.25 | 24 |
| ML2 | 1.28 | 18 |
| ML3 | 1.31 | 23 |
| Lac1 | 1.33 | 7 |
| Lac2 | 1.34 | 7 |
| ML4 | 1.59 | 43 |
| Flat curve | 2.64 | 870 |
| Gly | 3.56 | 4.5 |
| Myo-I | 3.49-3.52-3.54  3.60-3.62-3.65 | 5-8-7  6-6.5-4 |
|  |  |  |
| 2D COSY |  |  |
|  |  |  |
| PC | 3.60-4.20 |  |
| GPC | 3.70-4.34 |  |
|  |  |  |
| Culture media |  |  |
|  | **δ (ppm)** | **Δν (Hz)** |
|  | **High field** |  |
| Lac1 | 1.327 | 1.57 |
| Lac2 | 1.345 | 1.57 |
| Thr1 | 1.323 | 1.63 |
| Thr2 | 1.339 | 1.63 |

**Table S1:** mean values of chemical shifts δ (ppm) and line-widths Δν (Hz) after deconvolution of signals utilized for quantification. Values were obtained from spectra of at least three different samples. The standard deviation was lesser than 0.01 ppm for chemical shift values and 10% for linewidths. Chemical shifts are referred to lactate methyl at 1.33 ppm in both cell and culture media spectra. Chemical shifts were assigned according to literature (Behar et al., 1994, Seeger et al., 2003; Luciani et al., 2009; Grande et al., 2010; Govindaraju et al., 2000; Willker et al., 1996)

**Figure S3**

**A**

4

3

2

1

1

2

3

4

4

3

2

1

**Time (days)**

**Time (days)**

**Time (days)**

**Time (days)**

1

2

3

4

**Line #163 – cell spectra**

*2.40*

**Saccharopine**

*1.70*

*1.80*

*1.90*

*2.00*

*2.10*

*2.30*

*(ppm)*

**Saccharopine**

**B**

**Control**

**Oligomycin treated**

**Line #163 – culture medium spectra**

**Oligomycin treated**

**Control**

*2.20*

*(ppm)*

*1.280*

*1.300*

*1.320*

*1.340*

*1.360*

*1.380*

*(ppm)*

**Lac**

**C**

**Figure S3.** (A) Fold change of cell number, examined by Cell Titer Blue Viability Assay, for #61, #74, #1 and #163 lines after oligomycin treatment; (B) saccharopine and (C) lactate signal regions from control and oligomycin treated #163 line spectra.

**Figure S4**

**A**

**Line #163**

*6.52*

*(ppm)*

*6.48*

*6.48*

*6.52*

*(ppm)*

**Fumarate**

**control**

**control**

**Etomoxir**

**treated**

**Oligomycin treated**

**B**

**Line #1**

**control**

*6.48*

*6.52*

*(ppm)*

*6.48*

*6.52*

*(ppm)*

**control**

**Etomoxir**

**treated**

**Oligomycin treated**

**Figure S4. (A**) and (B) fumarate signal from line # 163 and line # 1 spectra respectively after oligomicyn and etomoxir treatments.

**Supplemental Experimental Procedures**

1D 1H MRS spectra of GSCs and culture media were acquired with a 90° RF pulse, the number of scans (ns) was equal to 1000 (sufficient to obtain a good signal-to-noise ratio) for cell spectra while ns=4000 was used for culture media spectra. When indicated, a Lorentzian-Gaussian function was applied in the time domain, before Fourier transformation.

2D COSY spectra were acquired with a 90°-t1-90°-t2 pulse sequence and ns=32 for cell or ns=128 for culture media samples. Spectra were acquired as a matrix of 512x128 data points in time domain.

Chemical shifts were measured with respect to Lac methyl signal at 1.33 ppm in 1D and to Lac cross peak at 1.33-4.12 ppm in 2D COSY cell and culture media spectra. Water residual signal was suppressed by presaturation in 1D and 2D experiments. Measurement time was approximately 210 min (60 min for the 1D experiment and 150 min for the 2D experiment) for cell and 12 hours (200 min for the 1D experiment and 9 hours for the 2D experiment) for culture media samples. Cell viability, tested by the trypan blue exclusion method, was greater than 90% at the beginning of the preparation and greater than 80% at the end of the MRS measurements, in agreement with previous works (Palma et al. 2011, Guidoni et al. 2014). MRS parameters were obtained in at least three independent experiments and data are expressed as mean ± S.D. values.

For 1D cell and culture media spectra, signal integration was performed using the 1DWIN-NMR software (Bruker, Darmstadt, Germany). The error in integration is about 5%.For overlapping signals a deconvolution of 1D spectrum was performed before integration using the same software. A Gaussian–Lorentzian ratio equal to 1 for the line shape function was chosen. A quantitative analysis was performed by measuring the fitted peak areas.

In cell spectra the area of macromolecules (MM) signal at 0.89 ppm was used as reference for the intensities. Supplementary Figure S2 shows signal deconvolution details and Table S1 reports relative parameters for Mobile Lipid (ML) and Glycine (Gly) signals. Details for GalNAc, UDP, tCr and MM signal deconvolution are reported in Guidoni et al. 2014.

The signal of amino acids (aa, sum of valine, isoleucine and leucine) was taken as intensity reference in culture media spectra. Figure S2C shows lactate doublet deconvolution; relative parameters are reported in Table in Figure S2.

2D WIN-NMR software (Bruker, AG, Darmstadt, Germany) was used to perform cross peak integration. The size of each rectangle marking the area used for volume integration of 2D COSY cross peaks was optimized according to previous studies (Guidoni et al., Binesh et al.), covering the peak and avoiding superimposing neighboring peaks. The plane baseline was evaluated and subtracted from the integrals. 2D signal integrals were normalized to the intensity of Lys cross peak at 1.70–3.00 ppm. This peak was considered representative of the cellular mass according to Palma (Palma et al. 2011) and references therein indicated. Table S1 in Figure S2 reports chemical shifts for PC and GPC 2D cross peaks.

**Supplemental References**

Behar, L., Rothman, D.L., Spencer, D.D., Petroff, O.A.C. Analysis of macromolecule resonances in 1H NMR spectra of human brain (1994). Magn Reson Med 32, 294-302 .

Binesh N, Yue K, Fairbanks L, Thomas MA. Reproducibility of localized 2D correlated MR spectroscopy. Magn. Reson. Med. 2002; 48: 942-948.

Govindaraju, V., Young, K., Maudsley, A.A. Proton NMR chemical shifts and coupling constants for brain metabolites (2010). NMR Biomed. 13, 129–153.

Grande, S., Palma, A., Luciani, A.M., Rosi, A., Guidoni, L., Viti, V. Glycosidic intermediates identified in 1H MR spectra of intact tumour cells may contribute to the clarification of aspects of glycosylation pathways (2010). NMR Biomed. 24, 68–79.

Luciani, A.M., Grande, S., Palma, A., Rosi, A., Giovannini, C., Sapora, O., Viti, V., Guidoni, L. Characterization of 1H NMR detectable mobile lipids in cells from human adenocarcinoma (2009). FEBS J 276: 1333-1346.

Palma, A., Grande, S., Rosi, A., Luciani, A.M., Guidoni, L., Viti, V. 1H MRS can detect aberrant glycosylation in tumour cells a study of the HeLa cell line (2010). NMR Biomed. 24, 1099–1110.

Seeger, U., Klose, U., Mader, I., Grodd, W., Näegle T. Parameterized evaluation of macromolecules and lipids in proton MR spectroscopy of brain diseases. Magn Reson Med 49:19-28 (2003).

Willker, W., Engelmann, J., Brand, A., Leibfritz D. Metabolite identification in cell extracts and culture media by proton-detected 2D [1H, 13C] NMR spectroscopy (1996) . J Magn Res Anal 2, 21-32.
